# Supplementary figures and images for: Zika virus infects pericytes in the choroid plexus and enters the central nervous system through the blood-cerebrospinal fluid barrier
Source: PLoS Pathog. 2020 May 1;16(5):e1008204. doi: 10.1371/journal.ppat.1008204 (PMC7194358; doi:10.1371/journal.ppat.1008204)

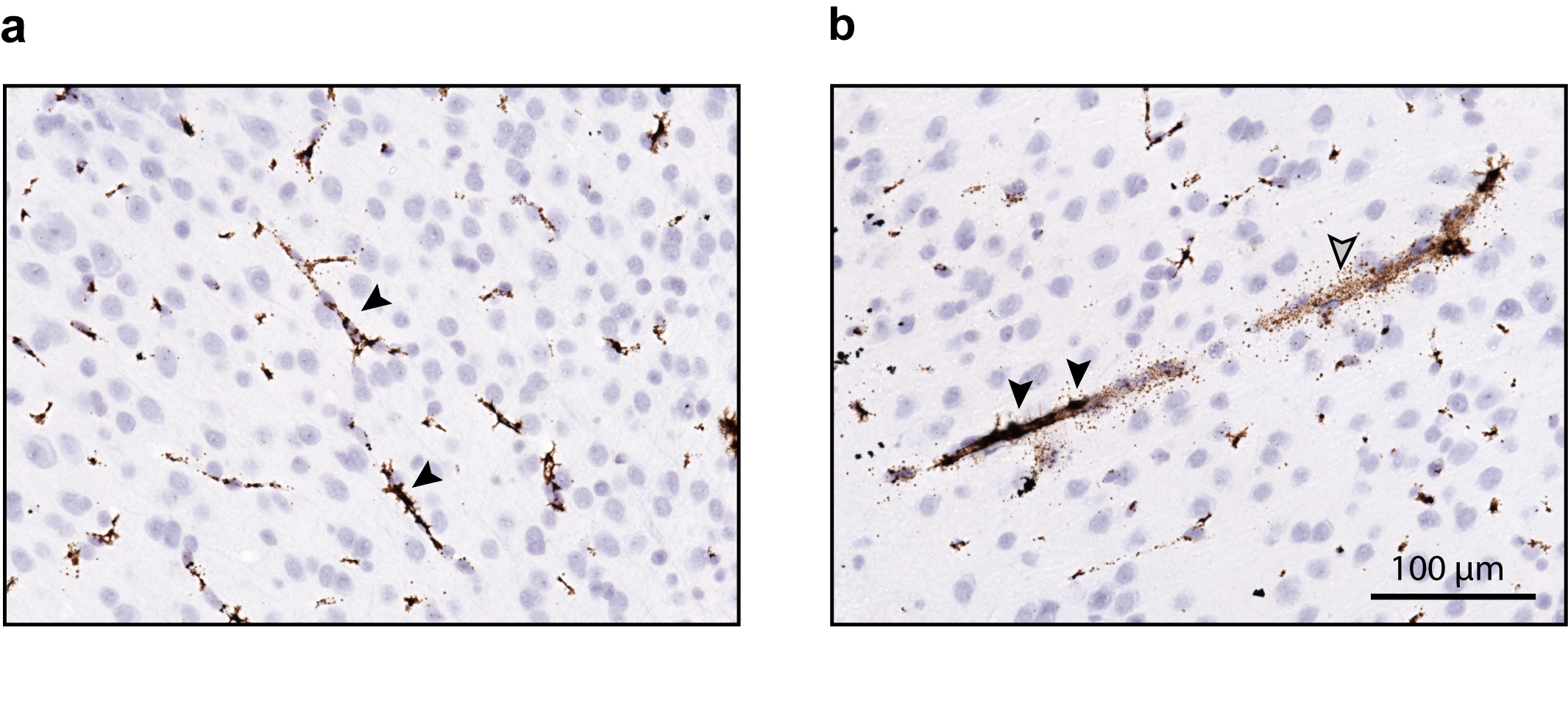

Supplement: S1 Fig — The brains infected with Venezuelan equine encephalitis virus (strain TC-83) showed widely distributed strong positive staining around the capillaries in the cortex. Representative images of viral RNA staining with RNAScope assay of the brain cortex of TC-83 infected AG129 mice (n = 6 from two independent experiments). Images were taken with a 40 x objective. Cortex capillaries showed strong positive straining (dark brown) for viral RNA. Black and gray arrowheads indicate virus-infected cells and virus -specific staining, respectively. (TIF) [file ppat.1008204.s001.tif]

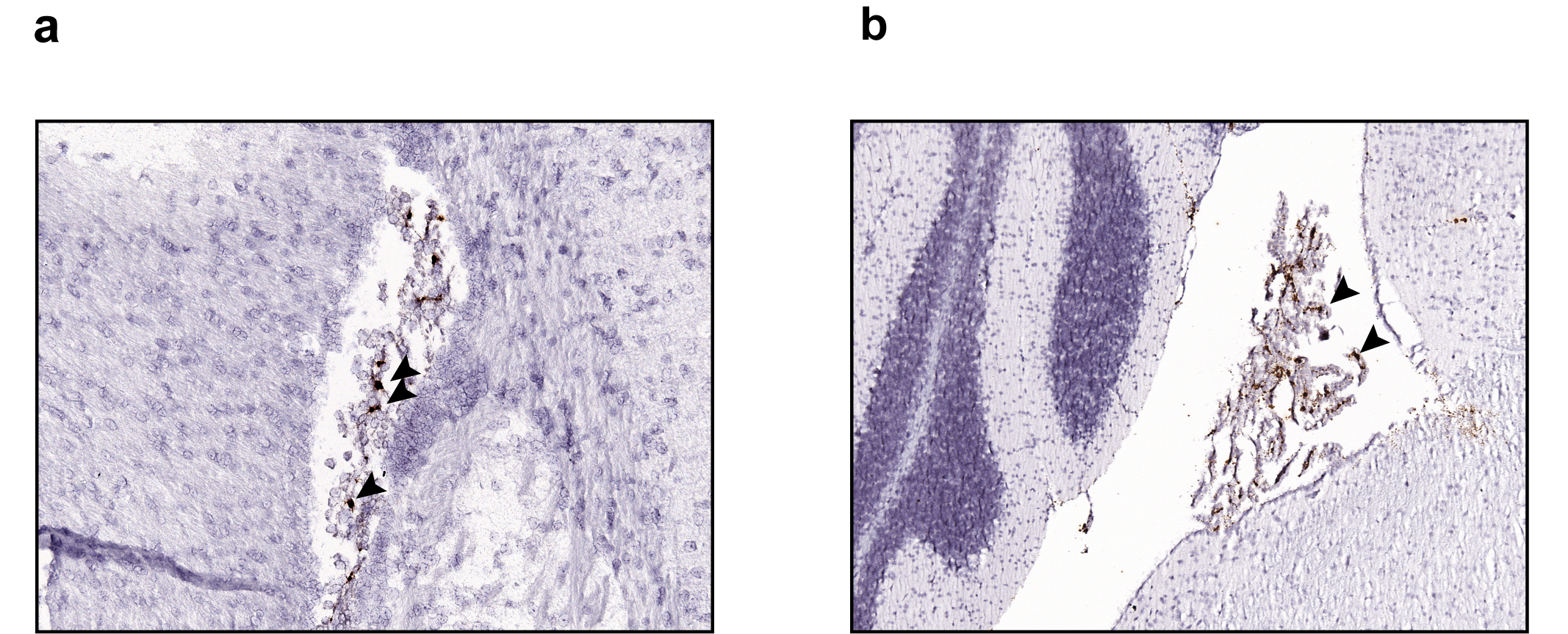

Supplement: S2 Fig — Ifnar-/- mice infected with the PLCal_ZV strain (n = 3, 1000 p.f.u./mouse) were euthanized at 4 d.p.i. and cardiac perfusion was performed with 25 mL of PBS to remove the blood prior to tissue harvest. Brains were subjected to RNAScope assay to detect viral RNA. Representative images were shown here. Black arrowheads indicate virus-infected cells in the lateral ChP (a) and 4th ventricle ChP (b). (TIF) [file ppat.1008204.s002.tif]

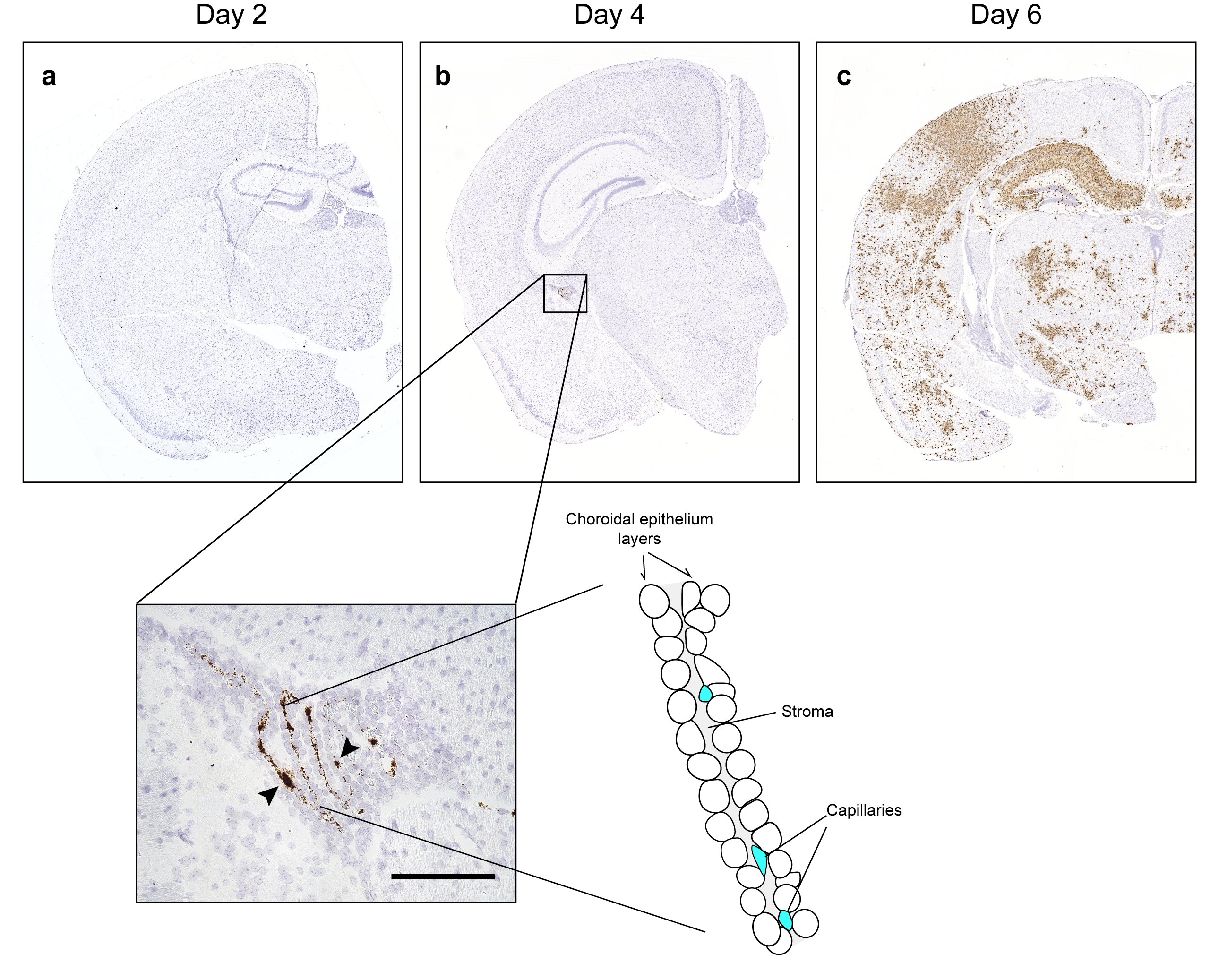

Supplement: S3 Fig — Brains from AG129 mice infected with PLCal_ZV (n = 3/timepoint, 1000 puf/mouse) were subjected to RNAScope assay to detect viral RNA. For a-c, representative images were shown from brains harvested at 2, 4, and 6 days post infection. Black arrowheads indicate virus-infected cells. Scale bars, 100 μm. (TIF) [file ppat.1008204.s003.tif]

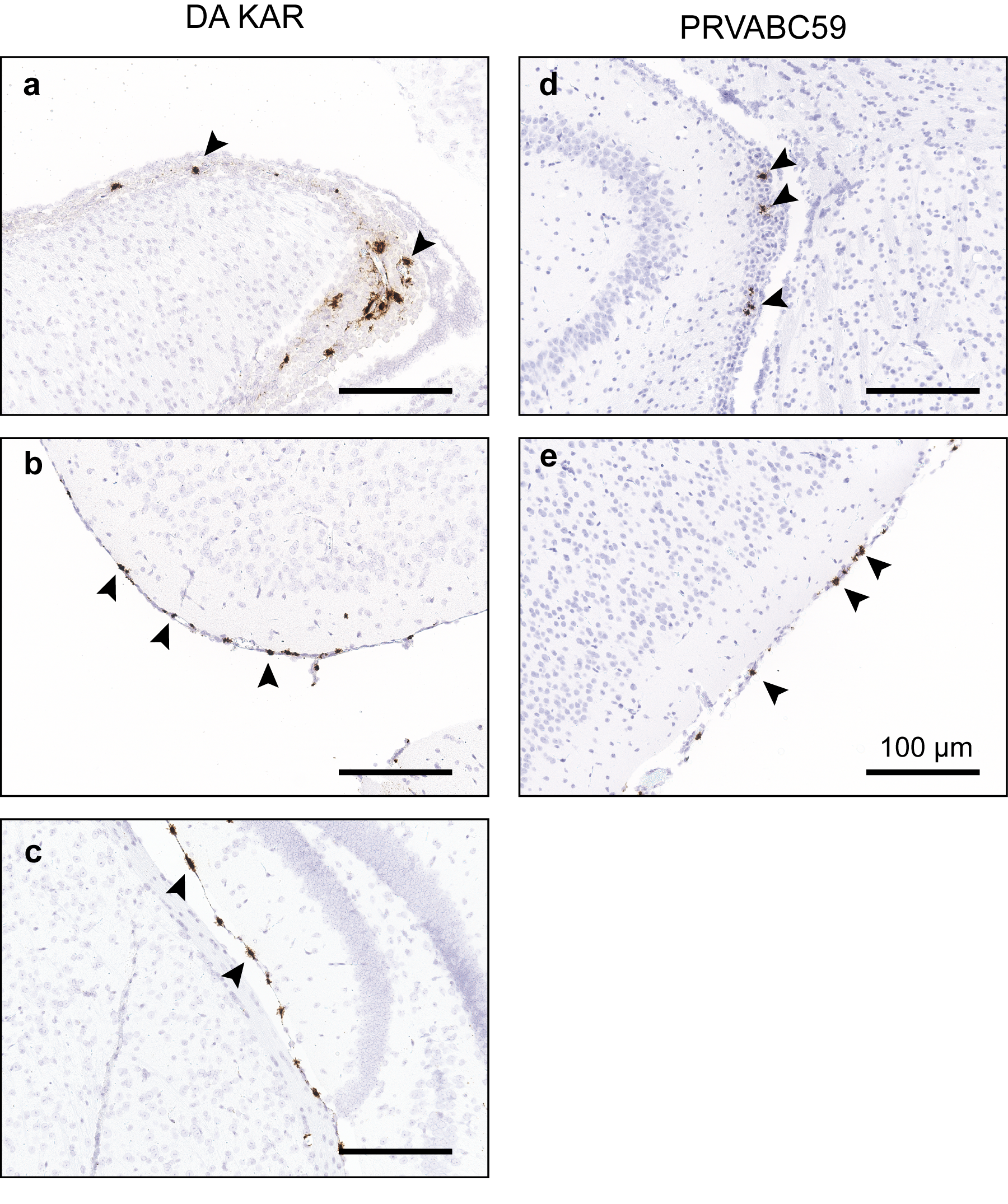

Supplement: S4 Fig — Representative images of the brains of AG129 mice infected with ZIKV strain DA KAR (a-c) and PRVABC59 (d-e) (n = 3 per group). The brains were harvested at 3 d.p.i. and were analyzed with RNAScope assay with a specific probe against the ZIKV. (TIF) [file ppat.1008204.s004.tif]

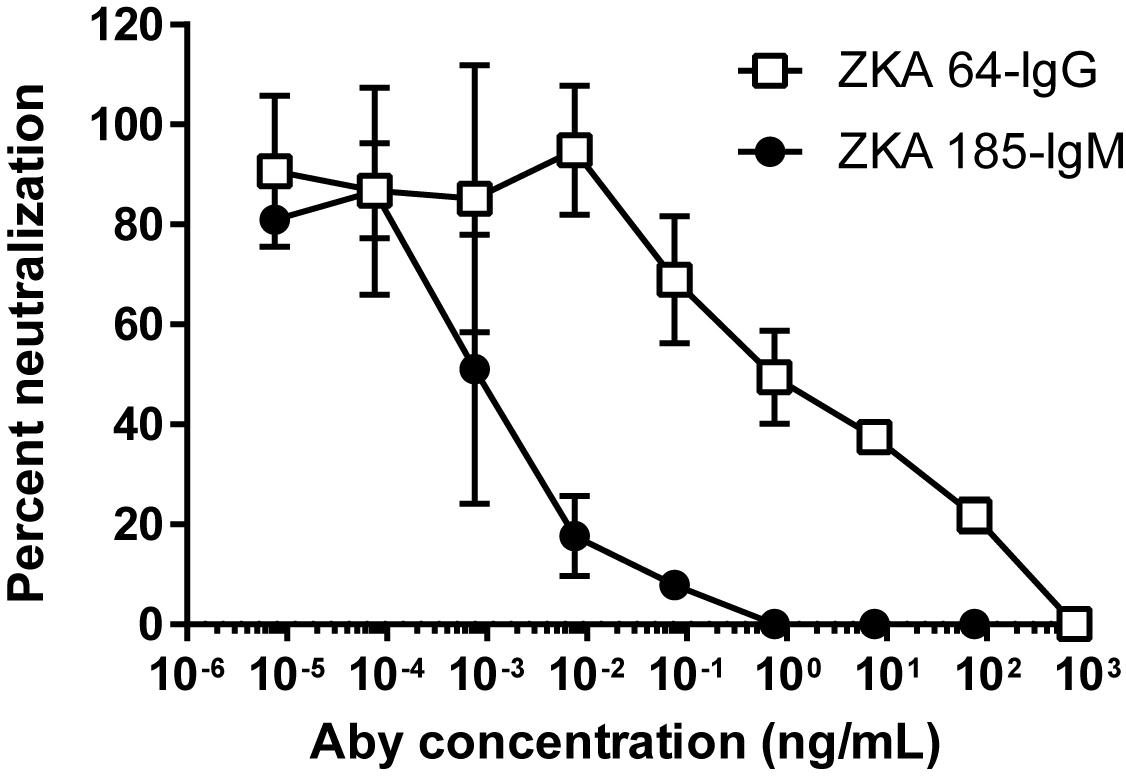

Supplement: S5 Fig — ZIKV-specific antibodies, clones ZKA 64 and ZKA 185, were serially diluted in cell growth media with HEPES (12.5 mM) and incubated with ZIKV strain PLCal_ZV (100 p.f.u./sample) for one hour at 37 °C. Vero 76 cells grown overnight in 12-well plates were infected with the antibody-virus mix and 5 days later viral plaques were developed by crystal violet staining. Anti-fluorescein mouse IgG and anti-fluorescein IgM were used as non-neutralizing antibody control (10 ng/mL). (JPG) [file ppat.1008204.s005.jpg]

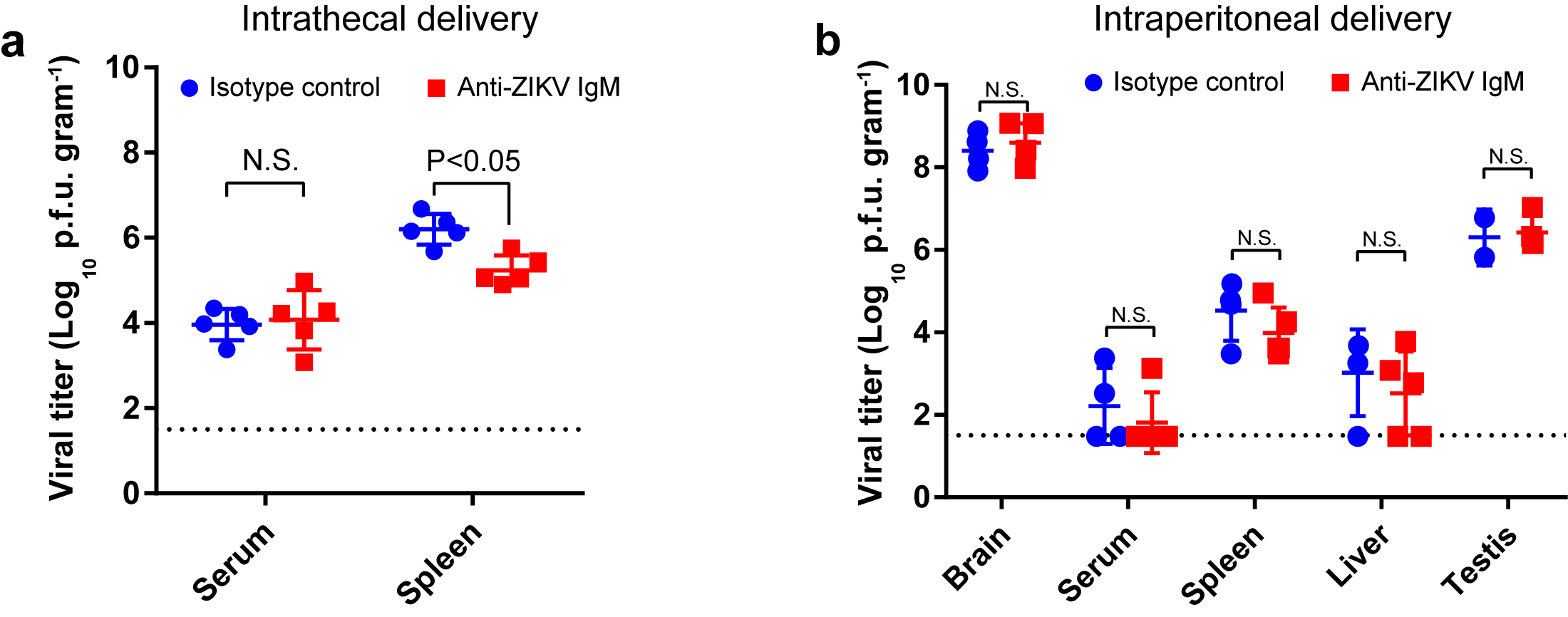

Supplement: S6 Fig — a, Intrathecal delivery of neutralizing antibody did not affect viral growth in peripheral tissues as much as in the brain. Viral loads of the serum and the spleens of ZIKV-infected mice treated either with isotype control (blue circles) or with ant-ZIKV IgM (red squares) showed no significant (serum) or less significant difference (spleen) than for the brains (6 d.p.i, n = 5-6/group). b, Intraperitoneal delivery of neutralizing antibody did not show any difference in viral replication in tissues, including brain. Antibodies (n = 4-5/group, 3 μg/mouse which is the same dose used for intrathecal delivery) were administrated intraperitoneally at 3 d.p.i. and the mice were euthanized at 7 d.p.i. Viral loads were determined with 10% tissue homogenates. N.S. no significance by Student t-test P> 0.05. (TIF) [file ppat.1008204.s006.tif]

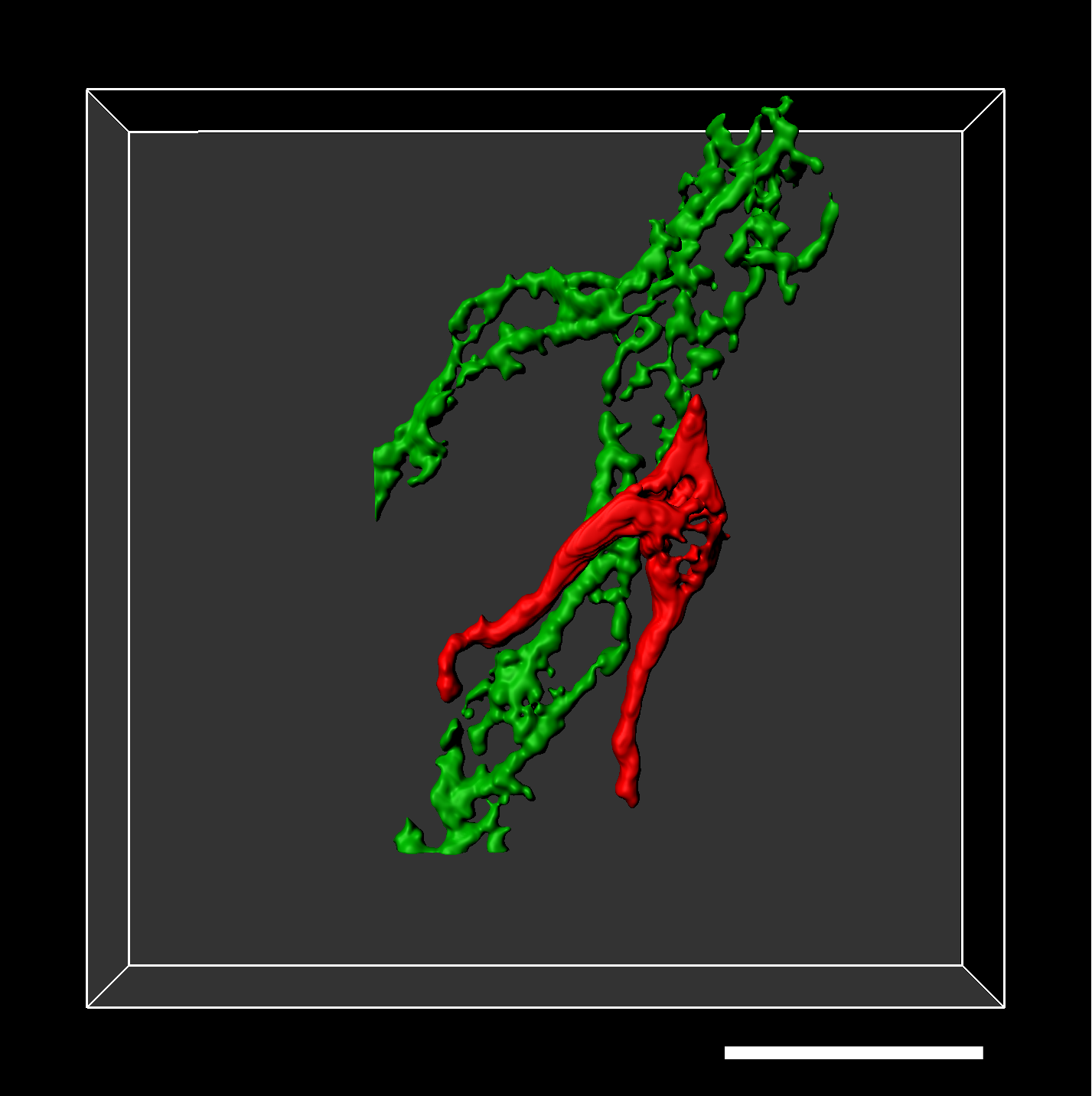

Supplement: S7 Fig — A reconstructed 3-D image of Fig 3b. Images were acquired with a confocal laser scanning microscope and a three-dimensional image was reconstructed with the image analysis software Imaris using its surface modeling function. CD31 (green) and ZIKV-E (red) were used as markers for endothelial cells and ZIKV-infected cells. Scare bar, 20 μm. (TIF) [file ppat.1008204.s007.tif]

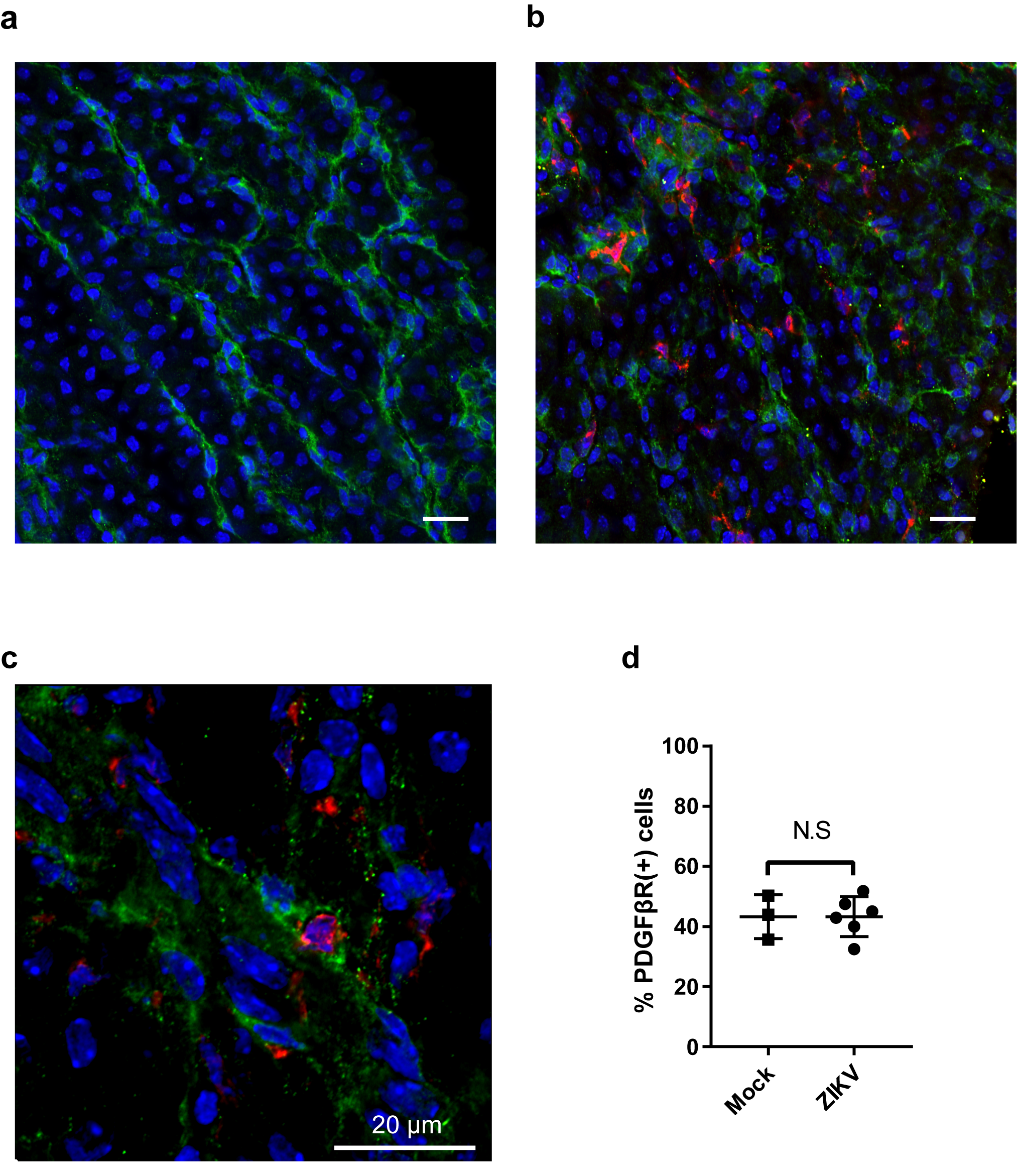

Supplement: S8 Fig — Mock (a) or ZIKV (b)–infected mice were euthanized at 4 dpi and cardiac perfusion was performed and the choroid plexuses were harvested. The whole-mount choroid plexus tissues were stained with rabbit anti-PDGFR-β (green, Alexa 488-conjucated anti-rabbit IgG) and mAby hu-4G2 (red, Alexa594-conjugated anti-human IgG) antibodies. Images of the stroma layer of the CPs were taken with Zeiss LSM 710Duo/Live5 confocal laser scanning fluorescence microscope with a 40 x object. c. A representative image with a high magnification (63X objective). d. Comparison of number of PDGFR-β(+) cells PDGFR-β(+) cells were counted from three mock-infected and six ZIKV-infected mouse choroid plexuses. N.S., P> 0.05 with Student t-test. (TIF) [file ppat.1008204.s008.tif]
